# The resistance mechanisms and treatment strategies for *EGFR*-mutant advanced non-small-cell lung cancer

**DOI:** 10.18632/oncotarget.20311

**Published:** 2017-08-17

**Authors:** Wen-Zhao Zhong, Qing Zhou, Yi-Long Wu

**Affiliations:** ^1^ Guangdong Lung Cancer Institute, Guangdong General Hospital and Guangdong Academy of Medical Sciences, Southern Medical University, Guangzhou 510080, China

**Keywords:** EGFR mutation, advanced NSCLC, EGFR-TKIs resistance mechanism, precision medicine

## Abstract

Epidermal growth factor receptor-tyrosine kinase inhibitors (EGFR-TKI) have been established as the standard therapy for *EGFR*-sensitizing mutant advanced non-small-cell lung cancer (NSCLC). However, patients ultimately develop resistance to these drugs. There are several mechanisms of both primary and secondary resistance to EGFR-TKIs. The primary resistance mechanisms include point mutations in exon 18, deletions or insertions in exon 19, insertions, duplications and point mutations in exon 20 and point mutation in exon 21 of *EGFR* gene. Secondary resistance to EGFR-TKIs is due to emergence of T790M mutation, activation of alternative signaling pathways, bypassing downstream signaling pathways and histological transformation. Strategies to overcome these intrinsic and acquired resistance mechanisms are complex. With the development of the precision medicine for advanced NSCLC, available systemic and local treatment options have expanded, requiring new clinical algorithms that take into account resistance mechanism. Though combination therapy is emerging as the standard of to overcome resistance mechanisms. Personalized treatment modalities based on molecular diagnosis and monitoring is essential for disease management. Emerging data from the ongoing clinical trials on combination therapy of third generation TKIs and antibodies in *EGFR* mutant NSCLC are promising for better survival outcomes.

## INTRODUCTION

The development of epidermal growth factor receptor (EGFR)-targeted tyrosine kinase inhibitors (TKI) has led to significant advances in patients with tumors harboring *EGFR* mutations (*EGFR*m). Approximately 50% of Asian patients with NSCLC have *EGFR* mutations [1]. In patients with sensitizing mutations of *EGFR*, first-line TKI treatment has good response rates of 50 to 80% compared to conventional chemotherapy that has response rate of 30%.Despite the demonstrated benefits of EGFR-targeting therapies, not all patients respond to treatment, moreover, patients who respond to EGFR-TKIs ultimately develop resistance to these drugs with the median progression-free survival (mPFS) around 9 to 13 months [2, 3]. Despite *EGFR* sensitizing mutations which show good response to EGFR TKIs, other *EGFR* mutations or pathways may play a role in development of resistance to EGFR TKIs. There are various mechanisms for development of resistance to EGFR TKIs. These include emergence of secondary mutation T790M, an amino acid substitution at position 790 in EGFR from a threonine to a methionine (T790M), bypass signaling activation , phenotype transition and aberrant downstream signaling pathways [4, 5]. Approximately 50% of patients who respond well initially to TKIs develop resistance due to the occurrence of secondary mutation T790M, This is the most common mechanism of acquired resistance to EGFR-TKIs [6, 7]. Till recently, no optimal therapy was available for NSCLC patients with T790M mutation in China. Hence, new clinical algorithms that take into account resistance mechanism and clinical pattern are warranted. The aim of this paper is to provide a comprehensive review of mechanisms of resistance to EGFR-TKIs and the potential treatment strategies to overcome this resistance in patients with advanced NSCLC.

### The resistance mechanisms of EGFR-TKI

#### Primary resistance

Primary, intrinsic or de novo resistance is defined as the failure to respond to the treatment at the first time after receiving TKI and presents no obvious symptoms improvement, disease control or overall survival (OS) [8, 9]. Primary resistance could be categorized into four classes: TKI resistance in the presence of a drug-sensitizing EGFR mutations, drug resistant EGFR mutations, genomic alterations along with EGFR mutations and EGFR wild-type tumors. The common mutations that confer primary resistance to TKIs are the result of non-sensitive EGFR mutation. Exon 19 mutations L747S/D761Y, exon 20 mutation T790M and exon 21 mutation T854A are some of the mutations that are associated with primary resistance [5]. Approximately 4% subgroup harbors insertions in *EGFR* exon 20 that account for intrinsic resistance [10]. *In vitro* studies showed that exon 20 `mutations were less sensitive to TKIs compared to exon 19 deletion and exon 21 point mutation (L868R) [11]. A concomitant T790M mutation in TKI naïve patients could also be the potential cause for intrinsic resistance [12]. Despite *EGFR*-related mutations, other mutations localized at the genes affecting EGFR downstream signaling might also lead to intrinsic resistance. Deletion of tumor suppressor gene *PTEN* or *PIK3CA* mutation can lead to aberrant activation of PI3K/AKT pathway [13, 14]. Other mechanisms, the crosstalk of EGFR and IGF1R pathway , the activation of NFκB pathway the polymorphisms of pro-apoptotic protein BIM, are also considered as the potential mechanisms for intrinsic resistance [15–17]. Increased expression of HGF, increases binding of MET-mediated activation of the PI3K–AKT pathway, thus hindering the ability of an EGFR TKI to inhibit this signaling pathway. The role of MET in primary resistance owing to increased HGF activation of MET is through GAB1 [21, 22]. IPASS clinical trial demonstrated insensitivity to gefitinib in tumors negative for EGFR mutations [24]. Studies have also reported role of *KRAS* mutations, *BRAF* mutations, *ALK* translocation and overexpression of HGF in primary resistance to EGFR TKIs [18–21]. BRAF mutations are present in approximately 2–3% EGFR wild-type tumor also confer intrinsic resistance due to the presence of other somatic mutations in genes encoding signaling molecules [19, 23]. Approximately 5% of tumors harbor ALK translocations that are reported to be insensitive to EGFR TKIs [20, 25].

#### Acquired resistance

A clinical definition of acquired resistance to EGFR TKIs has been proposed that is systemic progression (by RECIST or WHO criteria) after a complete or partial response or ≥ 6 months of stable disease after treatment with a targeted therapy [26, 27].

#### Secondary mutations of EGFR gene

The secondary mutations of *EGFR* gene that cause EGFR-TKI acquired resistance include T790M (exon 20), L747S (exon 19), D761Y (exon19) and T854A (exon 21) [28–30]. The most common mechanism of acquired resistance is due to emergence of T790M mutation, approximately 50% of NSCLC patients develop resistance to TKIs [31, 32]. Potential mechanism by which T790M causes resistance to EGFR-TKIs is by increasing the affinity to ATP [9, 33]. The mutation, results in substitution of threonine to methionine at the “gatekeeper” amino acid 790, leading to a bulkier side thus causing TKI medications to lose potency; This causes steric hindrance and the affinity of ATP at EGFR kinase domain recovers, which may lead to kinase phosphorylation and thereby activating the signaling pathways associated with tumor progression [34].

#### Bypass signaling activation

The tumor enhancing effects caused by *EGFR*-sensitizing mutations has impact on the activation of downstream signaling pathway [35]. Despite blocking the downstream signaling pathways by EGFR-TKI, other signaling pathway, such as *c-Met*, could initiated and facilitate tumor progression [36]. Several studies reported approximately 5–20% subsets harboring *c-Met* gene amplification in TKI resistant NSCLC patients [7, 37–39]. Additionally, *c-Met* gene amplification can be an independent to T790M to confer resistance to EGFR-TKIs[40]. *c-Met* gene amplification activates ERBB3 and thus activates the downstream PI3K/AKT pathway causing gefitinib and erlotinib resistance [41]. Therefore *c-Met* gene amplification could bypass the inhibited EGFR phosphorylation kinase pathway and activate downstream signal transduction, thus facilitating the tumor cells proliferation and developing resistance to EGFR-TKI. Other mechanisms of TKI resistance include *Her-2* amplification *MAK* amplification and *BRAF* (G469A and V600E)mutation [18, 42–43], *Her-2* amplification is reported in 10–20% of NSCLCs [44–47] and BRAF mutation is reported in 1–4% of NSCLCs [23, 48–50].

#### Phenotype transition

Phenotype transition includes transition to small cell lung cancer (SCLC) and epithelial mesenchymal transition (EMT). Sequist *et al.* found that 14% advanced NSCLC patients harboring *EGFR*-sensitizing mutations transformed into SCLC after TKI treatment [38]. Similar studies demonstrated SCLC transformation after TKI treatment in advanced NSCLC patients harboring *EGFR*-sensitizing mutations [51–53]. Therefore, it can be speculated that NSCLC transforming into SCLC might be one of the mechanisms of acquired EGFR-TKI and moreover, these SCLC also harbors similar EGFR mutations prior to transformation. In addition, Uramoto *et al.* indicated that about 40% (4/9) of patients with EGFR-TKI resistance presented epithelial cell transformation to mesenchymal cell, thus forming tumor stem cells, reducing the dependence on EGFR signaling pathway, and eventually leading to tumor malignance progression or metastasis [54].

#### Other causes

Several other EGFR-TKI resistance mechanisms have been identified including: downstream signaling pathway activation, such as the overexpression of insulin-like growth factors-1 receptor (IGF-1R). TKI could induce the formation of heterodimer of EGFR and IFG-1R in NSCLC cellular membrane, activate IFG-1R and its downstream signal transduction (PI3K/Akt), enhance anti-apoptosis effect and hence develop resistance to TKI [55].

### Treatment strategies for EGFR-TKI resistance

#### Treatment strategies for intrinsic EGFR-TKI resistance

Based on the mechanisms of EGFR intrinsic resistance caused by the concomitant gene mutations, combination therapy may be one potential solution. The combination of EGFR-targeted inhibitors and IGFIR inhibitors could potentially overcome resistance [56]. In mutant *EGFR* NSCLC patients, BCL-2 antibody triggers TKI-mediated apoptosis improving TKI efficacy [57, 58]. In patients with early disease stage (resected stage II-IIIA) with EGFR mutated NSCLC adjuvant chemotherapy/TKI treatment or both could be considered as an effective treatment. In patients with chemotherapy-naïve advanced EGFR mutated NSCLC chemotherapy or TKI treatment before after chemotherapy or concurrently could be an effective treatment [59]. In patients with late stage disease combination therapy has shown to be beneficial to prevent or delay emergence of resistance. Similarly, combination therapy is also seen to be effective in patients with other genomic alterations occurring with EGFR mutations [60, 61].

### Treatment strategies for acquired resistance

#### Treatment strategies based on clinical assessment

Yang *et al.* classified tumor progression after EGFR-TKI failure into three modes as gradual progression, local progression, and dramatic progression based on radiological and clinical examination results [62]. Disease control (lasting ≥ 6 months) with no significant increment in tumor burden in comparison with earlier assessment and asymptomatic status of pre-existing item (Symptom scored ≤ 1) with EGFR-TKI treatment is defines as gradual progression [63–65]. Disease control lasting ≥ 3 months with EGFR-TKI treatment with PD due to solitary extracranial lesion or limitation in intracranial lesions and asymptomatic status or stability of pre-existing item (Symptom scored ≤ 1) is defined as local progression [66–70]. Disease control (lasting ≥ 3 months) with rapid progression of multiple targeted lesions compared to previous assessment, or progressive involvement of non-target lesions with a score > 2 along with deterioration of any pre-existing item or new item (Symptom scored 2) after TKI treatment is defined as dramatic progression [64–66]. Yang et al. classification of clinical modes of EGFR-TKI failure could plausibility be beneficial in planning treatment modalities and predicting survival benefits in management of these patients [62].

In patients with gradual progression failing EGFR-TKI treatment, continuation of EGFR-TKI is recommended as TKI treatment after progression also showed good median PFS (14.9) months and could delay salvage therapy [71]. In patients with local progression, it is recommended to continue TKI therapy in conjunction with local therapy [62]. Yu *et al.* reported median OS up to 41 months in combination therapy of local therapy (surgical resection, radiofrequency ablation, or chemotherapy) combined with EGFR-TKI treatment after acquired resistance to EGFR-TKIs [72]. Gomez et al. reported improved progression free survival with local consolidated therapy with or without maintenance therapy compared to maintenance therapy alone [73]. However, there is still no sufficient evidence proving that local therapy in conjunction with EGFR-TKI is superior to local therapy alone or EGFR-TKI mono-therapy, respectively. In patients with dramatic progression, it is not recommended to continue TKI medications. As histological transformation to small-cell lung carcinoma occurs during active treatment with targeted therapy, in dynamic progression it is recommended to do biopsy to capture these histological and phenotypic changes as this could impact the chemotherapy options [38, 74–75]. Few studies suggest to continue chemotherapy after patients acquiring resistance [76, 77]. Kuo *et al.* reported higher remission rate (48.7% *vs* 21.4%), longer medium PFS (5.1 months *vs* 1.8 months) and medium OS (12.7 months *vs* 7 months) after receiving taxane-based chemotherapy than patients in nontaxane regimen [76] IMPRESS, a phase 3 randomized trial showed that progression-free survival was not prolonged in patients on continuation of gefitinib after disease progression compared to platinum-based doublet chemotherapy suggesting the later to be an gold-standard in such settings [78]. Nevertheless, more prospective studies are warranted for evaluating optimum chemotherapy strategy in this subset of patients.

Gandara et al. classified progressive disease (PD) into three categories as 1) CNS Sanctuary PD 2) Oligo-PD and Systemic PD in the settings of acquired resistance to EGFR-TKIs. These subtyping has implications in clinical settings and patient management [79]. In CNS sanctuary PD local therapy (surgical resection, focused radiotherapy or whole brain radiotherapy,) with continuation of TKIs is recommended. This approach had implications in extending PFS in this subtype of patients as reported by Gomez et al. in an randomized controlled trial and observational studies. [73, 80–81]. In oligo-PD a similar approach of treatment is seen to be beneficial with combination of local therapy and continuation of TKIs [73, 81]. In systemic PD several treatment strategies are available which include switching therapy or continuing with the same TKI to anticipation of slowing PD and continuation of the same TKI with addition of other therapies [3, 82–83].

### Targeted therapy based on acquired resistance mechanisms

#### Treatment strategies targeting secondary EGFR mutations

With the development of molecular biology, molecular targeted therapy based on the molecular mechanism of EGFR-TKI and targeted diverse resistance mechanisms has become the new direction fir advanced NSCLC precision medicine.

Emergence of *EGFR* T790M mutation is the preliminary mechanism of the acquired TKI resistance to the first-generation EGFR-TKIs (erlotinib and gefitinib). Although, the second-generation EGFR-TKIs (afatinib and dacomitinib) showed activity overcoming T790M mutation resistance in *in vitro* studies, clinical studies had disappointing results. Studies showed second-generation EGFR-TKI medications (afatinib and dacomitinib) had a remission rate lower than 10% in patients with TKI resistant advanced NSCLC [84, 85]. Since the efficacy of second-generation TKI monotherapy was not satisfactory, combination therapy has been explored. Janjigian *et al.* found that afatinib combined with cetuximab in treating EGFR-TKI resistant NSCLC patients harboring *EGFR*-sensitizing mutations could achieve an ORR of 29% and PFS of 5.7 months. However, the ORR between T790M-positive and T790M-negative patients was similar (32% vs 25%, P=0.341) and the overall efficacy was far from satisfaction, and the rate of side effects was high in combination medications, with the most common being rash (90%), diarrhea (71%), and stomatitis (56%) [86]. In two more studies, the combination therapy of cetuximab with erlotinib or gefitinib had no effect on OR [87, 88].

Compared to the second-generation TKIs, the third-generation EGFR-TKI selectively target T790M mutations, and irreversibly bind to EGFR ATP pocket, inhibiting EGFR kinase phosphorylation and the activation of downstream tumor signaling pathway. To date, the third-generation EGFR-TKI medications includes Osimertinib (AZD9291), CO-1686, HM61713, EGF816, ASP8273 and China local Avitinib [89–92]. Osimertinib (AZD9291) is small molecule, with high selective and irreversible suppressive effects on both NSCLC *EGFR*-sensitizing and T790M resistance mutations [89]. Osimertinib was approved for the treatment of advanced NSCLC patients with *EGFR* T790M mutations and acquired TKI resistance by FDA in November, 2015 [93]. In 2016, Osimertinib was further approved by EMA for the treatment of locally advanced or metastasis NSCLC patients with *EGFR* T790M mutation [94]. Recently, osimertinib has been approved in China by China food and Drug administration (CFDA) for metastatic or locally advanced NSCLC with T790M mutation [95]. Osimertinib has been approved for clinical usage in USA, EU, Japan, Canada, Switzerland, Israel, Korea and Mexico, mainly based on two AURA clinical trials [96–99]. In these two trials, a total of 411 patients with first-generation TKI resistance and EGFR T790M mutation were administrated with Osimertinib at the dosage of 80 mg/d. Patients with T790M mutations demonstrated response rate of 66–71% and median PFS of 9.6–10.1 months. For these patients, the median PFS in standard chemotherapy is estimated to be less than half a year [96, 97]. The preliminary data on osimertinib as first-line treatment in EGFR mutated treatment naïve NSCLC patients showed median progression free survival of 19.3 months (95% CI, 13.7, Not Calculable) with disease control rate of 97% (95% CI 88.5–99.6) and progression free survival in 55% of patients at 18 months with manageable safety profile [100]. The ongoing FLAURA (NCT02296125), a randomized, double-blind phase III trial to assess the efficacy and safety of osimertinib versus erlotinib/gefitinib as first-line therapy in EGFR mutated locally advance NSCLC are much awaited to position osimertinib as frontline therapy [101]. TATOON (NCT02143466), a multi-arm, phase 1b, study assessing the safety and tolerability of osimertinib with durvalumab versus osimertinib monotherapy has been suspended on the grounds of safety due to increased incidence of interstitial lung disease [102, 103]. In contrast, another third-generation EGFR-TKI, CO-1686, that had selective potency to T790M showed ORR of 28–34% in T790M positive patients [104]. However, CO-1686 and HM6171 are withdrawn from development due to poor efficacy outcomes [105, 106]. In addition, preliminary data on HM61713 revealed an ORR of 62% in T790M positive patients [107]. Other third-generation TKIs EGF816, ASP8273 and Avitinib are still in different stages of clinical development. Third-generation TKIs impact on clinical outcomes in EGFR mutated NSCLC patients are summarised in Table 1. Long-term follow-up studies are warranted to determine the efficacy of these third-generation EGFR-TKI.

**Table 1:** Third generation TKIs impact on clinical outcomes of EGFR T790M mutation positive and negative NSCLC patients

Study group	Treatment strategy (TKI-naïve/post-TKI)	Treatment	T790M positive				T790M Negative			
			ORR	DCR	Median PFS	Other outcomes	ORR	DCR	Median PFS	Other outcomes
Janne *et al.* [72]	Post TKI	Osimertinib (AZD9291)	61%	95%	9.6 months	−	21%	61%	6 months	−
Oxnard GR *et al.* [73]	Post TKI	Osimertinib (AZD9291)	62%	−	9.7 months	−	26%	−	3.4 months	−
Park *et al.*, [78]	Post TKI	HM61713	62%	91%	−	−	−	−	−	−
Yang J *et al.*, [74]	Post TKI	Osimertinib (AZD9291)	71%	−	9.7 months	DoR: 9.6 months	−	−	−	−
Wu YL et al, [81]	Post TKI	Avitinib (AC0010)	44%	85%	−	−	−	−	−	−

‘−’: not reported.

### Treatment strategies targeted at non-EGFR related secondary mutations

Besides T790M mutation, there are other mechanisms accounting for the resistance in nearly half of lung cancer patients, i.e. *c-Met* amplification, *Her-2* amplification, *PIK3CA* mutations and *BRAF* mutations. For these patients, combination therapy of inhibiting EGFR pathway together with bypass pathways signaling might overcome the acquired resistance. A phase II study investigated the therapeutic efficacy of a cMET inhibitor, INC280 or Capmatinib in combination with gefitinib for NSCLC patients with EGFR-TKI resistance and *cMET* positive, which demonstrated ORR of 18% (12/65) and DR of 62% (40/65) [108]. Neal *et al.* reported a phase II study combining Cabozantinib (MET inhibitor) and Erlotinib showed a longer median PFS, as compared with Erlotinib monotherapy (3.9 month vs 1.9 month) [109]. In addition, a Phase I clinical trial showed that the combination therapy of Erlotinib and PI3K inhibitor (BKM120) was well tolerated in advanced NSCLC patients with *EGFR*-sensitizing mutations and acquired resistance [110].

Cisplatin and etoposide-based therapy could be considered in patients with phenotypic modulation to SCLC [111]. The third-generation EGFR-TKI also somehow demonstrated efficacy in acquired-resistant patients with unclear mechanism. AURA phase-I trial indicated that Osimertinib had certain efficacy in T790M mutation-negative NSCLC patients with EGFR-TKI resistance, the subgroup at the dosage of 120mg/d demonstrating a 30% response rate. However, it should be noted that there are significant individual variations [112]. This might be attributed to tumor heterogeneity and the technical limitations in detecting T790M mutations. Different mechanisms of acquired resistance might occur in different specimens from the same *loci*, or from the different *loci*. Furthermore, even if for the potential T790M mutation-positive clone, when the concentration is lower than detection level, the result could show as negative. Hence, in AURA Phase I study, the demonstrated efficacy of Osimertinib in TKI resistant patients without EGFR T790M mutations might actually because of the undetectable T790M mutation clones. Therefore, it is still controversial regarding the effect of Osimertinib on TKI resistance induced by non-T790M mutations. Moreover, IMPRESS study showed that combination treatment of gefitinib and chemotherapy (cisplatin and pemetrexed) resulted in a PFS of 6.7 months in advanced NSCLC patients with TKI resistance T790M mutation negative patients [113]. Large scale studies are warranted to study the continuation of first-generation TKI in conjunction with chemotherapy which might be an alternative option for this subset of patients with *EGFR*-T790M mutation TKI resistant patients.

### Diagnosis and monitoring of EGFR-TKI resistance

Lung cancer management guidelines recommend molecular testing as a standard of care in patients with NSCLC [114–117]. As mutations, play an important role in determining personalized treatment, molecular mechanisms underlying these mutations is essential. Due to the intrinsic and acquired resistance mechanisms to EGFR-TKIs, dynamic monitoring of these mutations will be beneficial for early diagnosis and administration of effective drugs to overcome these resistance mechanisms and improve survival outcomes. Repeat biopsy is not feasible for monitoring disease and treatment response due to its limitations in terms of practicality, invasiveness, risk, availability of tissue, heterogeneity of tissue and advanced disease condition. Circulating tumor DNA (ctDNA) has emerged as a biomarker for real-time monitoring due to its less invasiveness, practicality and accessibility [118–120]. Several studies have shown concordance between tissue and blood based testing [118, 119, 121]. In sight of FDA approval for blood based testing using cobas *EGFR* mutation test for detecting EGFR mutations, it is practically possible to provide personalized treatment [122, 123]. Dynamic monitoring of T790M mutation plays an important role in patient management by early administration of third generation TKIs.

It is reported that the T790M mutation is detectable in plasma much earlier than the clinical manifestation of disease progression (PD) which is aided by dynamic monitoring of T790M mutation. Lee *et al.* monitored *EGFR* mutations during gefitinib or erlotinib treatment in plasma samples (N=367) revealed T790M detection rate of 28.6% in patients with PD. Among these 16.3% patients were detected with T790M mutation 2–12 months before PD and 12.2% during PD suggesting the importance of serial monitoring using plasma especially in patients with TKI resistance [124]. Similarly another study evaluated the clinical outcomes using quantification and dynamic monitoring of T790M mutation in plasma (*N* = 117) showed mutations in 47% of patients who developed TKI resistance (first or second line TKI) and in half of these positive patients the median time of early detection was 2.2 months before PD. Patients with detectable T790M showed lower OS compared to T790M negative group (26.9 months vs NA), moreover this positivity was associated with poor OS outcomes as identified by analysis of univariate and multivariate Cox proportional hazard model (HR = 1.716, 95% CI:1.014–2.903, *P* = 0.0443) [125]. Marchetti *et al.* reported early detection (at 35 days) of T790M in plasma after initiating erlotinib in slow responders, indicating development of early resistance [126]. These studies emphasize the importance of dynamic monitoring in treatment response and planning treatment strategies in these resistant NSCLC patients.

### Present research and future perspective

With the wide application of the third-generation EGFR-TKIs, it is demanded to develop the strategies overcoming acquired resistance to the third-generation EGFR-TKIs. To date, some ongoing clinical trials are engaged to determine the superiority of the combination of the third-generation EGFR-TKIs (Osimertinib, CO-1686 or others) with other agents (antibodies to BCL-2, MET or MEK), to third-generation EGFR-TKIs monotherapy. Moreover, studies testing EGFR-TKIs combined with EGFR monoclonal antibody are also currently ongoing. Table 2 summarizes the clinical trials on combination therapy of EGFR-TKIs with antibodies for treatment of NSCLC. These studies would spread the lights on the treatment of EGFR-TKI resistant NSCLC patients in the near future.

**Table 2:** Summary of clinical trials on combination therapy of EGFR-TKI with antibodies for treatment of NSCLC

Trial number	Indication/EGFR Mutation status	Phase	Drug	Treatment	Sponsor
NCT02454933	T790M positive advanced NSCLC	III	Osimertinib MEDI4736	Osimertinib And MEDI4736 Vs Osimertinib monotherapy	AstraZeneca
NCT02143466	Advanced EGFR mutation positive NSCLC	I	Osimertinib AZD6094 MEDI4736	Osimertinib and AZD6094 Osimertinib and selumetinib Osimertinib and MEDI4736 Vs AZD6094 monotherapy	AstraZeneca
NCT02789345	Advanced T790M mutation positive NSCLC	I	Ramucirumab Necitumumab Osimertinib	Ramucirumab or Necitumumab in combination with Osimertinib	Eli Lilly and Company
				A	
NCT02040064	EGFR mutant NSCLC	I	Gefitinib Tremelimumab	Gefitinib and Tremelimumab	Gustave Roussy, Cancer Campus, Grand Paris
NCT03054038	Advanced EGFR mutant NSCLC	I	Afatinib Necitumumab	Afatinib and Necitumumab	Vanderbilt-Ingram Cancer Center
NCT02716311	EGFR mutated NSCLC	II	Afatinib Cetuximab	Afatinib and Cetuximab	Intergroupe Francophone de Cancerologie Thoracique

## CONCLUSIONS

EGFR-TKI is the standard treatment for advanced NSCLC patients harboring *EGFR*-sensitizing mutations. However, intrinsic and acquired resistance is unavoidable. Currently the key treatment approach for EGFR-TKI resistance is to be based on EGFR-TKI resistance mechanism and clinical implications. However, due to tumor heterogeneity and the limitation of second biopsy, targeted at one single resistance mechanism might be insufficient. Therefore, the combination overcoming multiple resistance mechanisms is the new direction under the era of precision medicine.

## References

[1] Shi Y, Au JS, Thongprasert S, Srinivasan S, Tsai CM, Khoa MT, Heeroma K, Itoh Y, Cornelio G, Yang PC (2014). A prospective, molecular epidemiology study of EGFR mutations in Asian patients with advanced non-small-cell lung cancer of adenocarcinoma histology (PIONEER). J Thorac Oncol.

[2] Mok TS, Wu YL, Yu CJ, Zhou C, Chen YM, Zhang L, Ignacio J, Liao M, Srimuninnimit V, Boyer MJ, Chua-Tan M, Sriuranpong V, Sudoyo AW (2009). Randomized, placebo-controlled, phase II study of sequential erlotinib and chemotherapy as first-line treatment for advanced non-small-cell lung cancer. J Clin Oncol.

[3] Rosell R, Carcereny E, Gervais R, Vergnenegre A, Massuti B, Felip E, Palmero R, Garcia-Gomez R, Pallares C, Sanchez JM, Porta R, Cobo M, Garrido P (2012). Erlotinib versus standard chemotherapy as first-line treatment for European patients with advanced EGFR mutation-positive non-small-cell lung cancer (EURTAC): a multicentre, open-label, randomised phase 3 trial. Lancet Oncol.

[4] Huang L, Fu L (2015). Mechanisms of resistance to EGFR tyrosine kinase inhibitors. Acta Pharm Sin B.

[5] Pao W, Chmielecki J (2010). Rational, biologically based treatment of EGFR-mutant non-small-cell lung cancer. Nat Rev Cancer.

[6] Pao W, Miller VA, Politi KA, Riely GJ, Somwar R, Zakowski MF, Kris MG, Varmus H (2005). Acquired resistance of lung adenocarcinomas to gefitinib or erlotinib is associated with a second mutation in the EGFR kinase domain. PLoS Med.

[7] Yu HA, Arcila ME, Rekhtman N, Sima CS, Zakowski MF, Pao W, Kris MG, Miller VA, Ladanyi M, Riely GJ (2013). Analysis of tumor specimens at the time of acquired resistance to EGFR-TKI therapy in 155 patients with EGFR-mutant lung cancers. Clin Cancer Res.

[8] Wang J, Wang B, Chu H, Yao Y (2016). Intrinsic resistance to EGFR tyrosine kinase inhibitors in advanced non-small-cell lung cancer with activating EGFR mutations. OncoTargets Ther.

[9] Morgillo F, Della Corte CM, Fasano M, Ciardiello F (2016). Mechanisms of resistance to EGFR-targeted drugs: lung cancer. ESMO Open.

[10] Yasuda H, Kobayashi S, Costa DB (2012). EGFR exon 20 insertion mutations in non-small-cell lung cancer: preclinical data and clinical implications. Lancet Oncol.

[11] Greulich H, Chen TH, Feng W, Jänne PA, Alvarez JV, Zappaterra M, Bulmer SE, Frank DA, Hahn WC, Sellers WR, Meyerson M (2005). Oncogenic transformation by inhibitor-sensitive and -resistant EGFR mutants. PLoS Med.

[12] Yu HA, Arcila ME, Hellmann MD, Kris MG, Ladanyi M, Riely GJ (2014). Poor response to erlotinib in patients with tumors containing baseline EGFR T790M mutations found by routine clinical molecular testing. Ann Oncol.

[13] Sos ML, Koker M, Weir BA, Heynck S, Rabinovsky R, Zander T, Seeger JM, Weiss J, Fischer F, Frommolt P, Michel K, Peifer M, Mermel C (2009). PTEN loss contributes to erlotinib resistance in EGFR-mutant lung cancer by activation of Akt and EGFR. Cancer Res.

[14] Carcereny E, Molina MA, Sanchez JJ, Bertran-Alamillo J, Mayo C, Aldeguer E, Gimenez Capitan A, Yeste Z, Costa C, Benlloch S, Martinez A, Buges C, Bosch J (2011). Mutations of the catalytic subunit a of PI3K (PIK3CA) in erlotinib-treated non-small cell lung cancer (NSCLC) patients (p) with epidermal growth factor receptor (EGFR) mutations. J Clin Oncol.

[15] Lee Y, Wang Y, James M, Jeong JH, You M (2016). Inhibition of IGF1R signaling abrogates resistance to afatinib (BIBW2992) in EGFR T790M mutant lung cancer cells. Mol Carcinog.

[16] Lin K, Cheng J, Yang T, Li Y, Zhu B (2015). EGFR-TKI down-regulates PD-L1 in EGFR mutant NSCLC through inhibiting NF-κB. Biochem Biophys Res Commun.

[17] Huang WF, Liu AH, Zhao HJ, Dong HM, Liu LY, Cai SX (2015). BIM Gene Polymorphism Lowers the Efficacy of EGFR-TKIs in Advanced Nonsmall Cell Lung Cancer With Sensitive EGFR Mutations: A Systematic Review and Meta-Analysis. Medicine (Baltimore).

[18] Pao W, Wang TY, Riely GJ, Miller VA, Pan Q, Ladanyi M, Zakowski MF, Heelan RT, Kris MG, Varmus HE (2005). KRAS mutations and primary resistance of lung adenocarcinomas to gefitinib or erlotinib. PLoS Med.

[19] Brose MS, Volpe P, Feldman M, Kumar M, Rishi I, Gerrero R, Einhorn E, Herlyn M, Minna J, Nicholson A, Roth JA, Albelda SM, Davies H (2002). BRAF and RAS mutations in human lung cancer and melanoma. Cancer Res.

[20] Soda M, Choi YL, Enomoto M, Takada S, Yamashita Y, Ishikawa S, Fujiwara S, Watanabe H, Kurashina K, Hatanaka H, Bando M, Ohno S, Ishikawa Y (2007). Identification of the transforming EML4-ALK fusion gene in non-small-cell lung cancer. Nature.

[21] Yano S, Wang W, Li Q, Matsumoto K, Sakurama H, Nakamura T, Ogino H, Kakiuchi S, Hanibuchi M, Nishioka Y, Uehara H, Mitsudomi T, Yatabe Y (2008). Hepatocyte growth factor induces gefitinib resistance of lung adenocarcinoma with epidermal growth factor receptor-activating mutations. Cancer Res.

[22] Turke AB, Zejnullahu K, Wu YL, Song Y, Dias-Santagata D, Lifshits E, Toschi L, Rogers A, Mok T, Sequist L, Lindeman NI, Murphy C, Akhavanfard S (2010). Preexistence and clonal selection of MET amplification in EGFR mutant NSCLC. Cancer Cell.

[23] Naoki K, Chen TH, Richards WG, Sugarbaker DJ, Meyerson M (2002). Missense mutations of the BRAF gene in human lung adenocarcinoma. Cancer Res.

[24] Mok TS, Wu YL, Thongprasert S, Yang CH, Chu DT, Saijo N, Sunpaweravong P, Han B, Margono B, Ichinose Y, Nishiwaki Y, Ohe Y, Yang JJ (2009). Gefitinib or carboplatin-paclitaxel in pulmonary adenocarcinoma. N Engl J Med.

[25] Shaw AT, Yeap BY, Mino-Kenudson M, Digumarthy SR, Costa DB, Heist RS, Solomon B, Stubbs H, Admane S, McDermott U, Settleman J, Kobayashi S, Mark EJ (2009). Clinical features and outcome of patients with non-small-cell lung cancer who harbor EML4-ALK. J Clin Oncol.

[26] Jackman D, Pao W, Riely GJ, Engelman JA, Kris MG, Jänne PA, Lynch T, Johnson BE, Miller VA (2010). Clinical definition of acquired resistance to epidermal growth factor receptor tyrosine kinase inhibitors in non-small-cell lung cancer. J Clin Oncol.

[27] Sacher AG, Jänne PA, Oxnard GR (2014). Management of acquired resistance to epidermal growth factor receptor kinase inhibitors in patients with advanced non-small cell lung cancer: Acquired Resistance to EGFR Kinase Inhibitors. Cancer.

[28] Balak MN, Gong Y, Riely GJ, Somwar R, Li AR, Zakowski MF, Chiang A, Yang G, Ouerfelli O, Kris MG, Ladanyi M, Miller VA, Pao W (2006). Novel D761Y and common secondary T790M mutations in epidermal growth factor receptor-mutant lung adenocarcinomas with acquired resistance to kinase inhibitors. Clin Cancer Res.

[29] Bean J, Riely GJ, Balak M, Marks JL, Ladanyi M, Miller VA, Pao W (2008). Acquired resistance to epidermal growth factor receptor kinase inhibitors associated with a novel T854A mutation in a patient with EGFR-mutant lung adenocarcinoma. Clin Cancer Res.

[30] Costa DB, Halmos B, Kumar A, Schumer ST, Huberman MS, Boggon TJ, Tenen DG, Kobayashi S (2007). BIM mediates EGFR tyrosine kinase inhibitor-induced apoptosis in lung cancers with oncogenic EGFR mutations. PLoS Med.

[31] Oxnard GR, Arcila ME, Chmielecki J, Ladanyi M, Miller VA, Pao W (2011). New strategies in overcoming acquired resistance to epidermal growth factor receptor tyrosine kinase inhibitors in lung cancer. Clin Cancer Res.

[32] Gazdar AF (2009). Activating and resistance mutations of EGFR in non-small-cell lung cancer: role in clinical response to EGFR tyrosine kinase inhibitors. Oncogene.

[33] Kirpichnikov VS, Faktorovich KA, Suleimanyan VS (1974). Increasing the resistance of carp to dropsy by breeding. I. Methods of breeding for resistance. Sov Genet.

[34] Yun CH, Mengwasser KE, Toms AV, Woo MS, Greulich H, Wong KK, Meyerson M, Eck MJ (2008). The T790M mutation in EGFR kinase causes drug resistance by increasing the affinity for ATP. Proc Natl Acad Sci USA.

[35] Zhang Y, Wang L, Zhang M, Jin M, Bai C, Wang X (2012). Potential mechanism of interleukin-8 production from lung cancer cells: an involvement of EGF-EGFR-PI3K-Akt-Erk pathway. J Cell Physiol.

[36] Sattler M, Hasina R, Reddy MM, Gangadhar T, Salgia R (2011). The role of the c-Met pathway in lung cancer and the potential for targeted therapy. Ther Adv Med Oncol.

[37] Preusser M, Streubel B, Berghoff AS, Hainfellner JA, von Deimling A, Widhalm G, Dieckmann K, Wöhrer A, Hackl M, Zielinski C, Birner P (2014). Amplification and overexpression of CMET is a common event in brain metastases of non-small cell lung cancer. Histopathology.

[38] Sequist LV, Waltman BA, Dias-Santagata D, Digumarthy S, Turke AB, Fidias P, Bergethon K, Shaw AT, Gettinger S, Cosper AK, Akhavanfard S, Heist RS, Temel J (2011). Genotypic and histological evolution of lung cancers acquiring resistance to EGFR inhibitors. Sci Transl Med.

[39] Ji W, Choi CM, Rho JK, Jang SJ, Park YS, Chun SM, Kim WS, Lee JS, Kim SW, Lee DH, Lee JC (2013). Mechanisms of acquired resistance to EGFR-tyrosine kinase inhibitor in Korean patients with lung cancer. BMC Cancer.

[40] Bean J, Brennan C, Shih JY, Riely G, Viale A, Wang L, Chitale D, Motoi N, Szoke J, Broderick S, Balak M, Chang WC, Yu CJ (2007). MET amplification occurs with or without T790M mutations in EGFR mutant lung tumors with acquired resistance to gefitinib or erlotinib. Proc Natl Acad Sci USA.

[41] Engelman JA, Zejnullahu K, Mitsudomi T, Song Y, Hyland C, Park JO, Lindeman N, Gale CM, Zhao X, Christensen J, Kosaka T, Holmes AJ, Rogers AM (2007). MET amplification leads to gefitinib resistance in lung cancer by activating ERBB3 signaling. Science.

[42] Takezawa K, Pirazzoli V, Arcila ME, Nebhan CA, Song X, de Stanchina E, Ohashi K, Janjigian YY, Spitzler PJ, Melnick MA, Riely GJ, Kris MG, Miller VA (2012). HER2 amplification: a potential mechanism of acquired resistance to EGFR inhibition in EGFR-mutant lung cancers that lack the second-site EGFRT790M mutation. Cancer Discov.

[43] Ohashi K, Sequist LV, Arcila ME, Moran T, Chmielecki J, Lin YL, Pan Y, Wang L, de Stanchina E, Shien K, Aoe K, Toyooka S, Kiura K (2012). Lung cancers with acquired resistance to EGFR inhibitors occasionally harbor BRAF gene mutations but lack mutations in KRAS, NRAS, or MEK1. Proc Natl Acad Sci USA.

[44] Hirsch FR, Varella-Garcia M, Franklin WA, Veve R, Chen L, Helfrich B, Zeng C, Baron A, Bunn PA (2002). Evaluation of HER-2/neu gene amplification and protein expression in non-small cell lung carcinomas. Br J Cancer.

[45] Pellegrini C, Falleni M, Marchetti A, Cassani B, Miozzo M, Buttitta F, Roncalli M, Coggi G, Bosari S (2003). HER-2/Neu alterations in non-small cell lung cancer: a comprehensive evaluation by real time reverse transcription-PCR, fluorescence *in situ* hybridization, and immunohistochemistry. Clin Cancer Res.

[46] Heinmöller P, Gross C, Beyser K, Schmidtgen C, Maass G, Pedrocchi M, Rüschoff J (2003). HER2 status in non-small cell lung cancer: results from patient screening for enrollment to a phase II study of herceptin. Clin Cancer Res.

[47] Mar N, Vredenburgh JJ, Wasser JS (2015). Targeting HER2 in the treatment of non-small cell lung cancer. Lung Cancer.

[48] Brose MS, Volpe P, Feldman M, Kumar M, Rishi I, Gerrero R, Einhorn E, Herlyn M, Minna J, Nicholson A, Roth JA, Albelda SM, Davies H (2002). BRAF and RAS mutations in human lung cancer and melanoma. Cancer Res.

[49] Cardarella S, Ogino A, Nishino M, Butaney M, Shen J, Lydon C, Yeap BY, Sholl LM, Johnson BE, Jänne PA (2013). Clinical, pathologic, and biologic features associated with BRAF mutations in non-small cell lung cancer. Clin Cancer Res.

[50] Paik PK, Arcila ME, Fara M, Sima CS, Miller VA, Kris MG, Ladanyi M, Riely GJ (2011). Clinical characteristics of patients with lung adenocarcinomas harboring BRAF mutations. J Clin Oncol.

[51] Piotrowska Z, Niederst MJ, Karlovich CA, Wakelee HA, Neal JW, Mino-Kenudson M, Fulton L, Hata AN, Lockerman EL, Kalsy A, Digumarthy S, Muzikansky A, Raponi M (2015). Heterogeneity Underlies the Emergence of EGFRT790 Wild-Type Clones Following Treatment of T790M-Positive Cancers with a Third-Generation EGFR Inhibitor. Cancer Discov.

[52] Kim TM, Song A, Kim DW, Kim S, Ahn YO, Keam B, Jeon YK, Lee SH, Chung DH, Heo DS (2015). Mechanisms of Acquired Resistance to AZD9291: A Mutation-Selective, Irreversible EGFR Inhibitor. J Thorac Oncol.

[53] Alam N, Gustafson KS, Ladanyi M, Zakowski MF, Kapoor A, Truskinovsky AM, Dudek AZ Small-cell carcinoma with an epidermal growth factor receptor mutation in a never-smoker with gefitinib-responsive adenocarcinoma of the lung. Clin Lung Cancer.

[54] Uramoto H, Iwata T, Onitsuka T, Shimokawa H, Hanagiri T, Oyama T (2010). Epithelial-mesenchymal transition in EGFR-TKI acquired resistant lung adenocarcinoma. Anticancer Res.

[55] Dong S, Qu X, Li W, Zhong X, Li P, Yang S, Chen X, Shao M, Zhang L (2015). The long non-coding RNA, GAS5, enhances gefitinib-induced cell death in innate EGFR tyrosine kinase inhibitor-resistant lung adenocarcinoma cells with wide-type EGFR via downregulation of the IGF-1R expression. J Hematol Oncol.

[56] Tandon R, Kapoor S, Vali S, Senthil V, Nithya D, Venkataramanan R, Sharma A, Talwadkar A, Ray A, Bhatnagar PK, Dastidar SG (2011). Dual epidermal growth factor receptor (EGFR)/insulin-like growth factor-1 receptor (IGF-1R) inhibitor: a novel approach for overcoming resistance in anticancer treatment. Eur J Pharmacol.

[57] Moretti L, Li B, Kim KW, Chen H, Lu B (2010). AT-101, a pan-Bcl-2 inhibitor, leads to radiosensitization of non-small cell lung cancer. J Thorac Oncol.

[58] Gong Y, Somwar R, Politi K, Balak M, Chmielecki J, Jiang X, Pao W (2007). Induction of BIM is essential for apoptosis triggered by EGFR kinase inhibitors in mutant EGFR-dependent lung adenocarcinomas. PLoS Med.

[59] Wu YL, Zhong W, Wang Q (2017). Gefitinib (G) versus vinorelbine+cisplatin (VP) as adjuvant treatment in stage II-IIIA (N1–N2) non-small-cell lung cancer (NSCLC) with EGFR-activating mutation (ADJUVANT): A randomized, Phase III trial (CTONG 1104). J Clin Oncol.

[60] Pao W, Wang TY, Riely GJ, Miller VA, Pan Q, Ladanyi M, Zakowski MF, Heelan RT, Kris MG, Varmus HE (2005). KRAS Mutations Primary Resistance of Lung Adenocarcinomas to Gefitinib or Erlotinib. PLoS Med.

[61] Wu Y, Yang J, Kim D (2014). Safety and efficacy of INC280 in combination with gefitinib (gef) in patients with EGFR-mutated (mut). MET-positive NSCLC: a single-arm phase lb/ll study. J Clin Oncol.

[62] Yang JJ, Chen HJ, Yan HH, Zhang XC, Zhou Q, Su J, Wang Z, Xu CR, Huang YS, Wang BC, Yang XN, Zhong WZ, Nie Q (2013). Clinical modes of EGFR tyrosine kinase inhibitor failure and subsequent management in advanced non-small cell lung cancer. Lung Cancer.

[63] Kaira K, Naito T, Takahashi T, Ayabe E, Shimoyama R, Kaira R, Ono A, Igawa S, Shukuya T, Murakami H, Tsuya A, Nakamura Y, Endo M (2010). Pooled analysis of the reports of erlotinib after failure of gefitinib for non-small cell lung cancer. Lung Cancer.

[64] Therasse P, Arbuck SG, Eisenhauer EA, Wanders J, Kaplan RS, Rubinstein L, Verweij J, Van Glabbeke M, van Oosterom AT, Christian MC, Gwyther SG (2000). New guidelines to evaluate the response to treatment in solid tumors. European Organization for Research and Treatment of Cancer, National Cancer Institute of the United States, National Cancer Institute of Canada. J Natl Cancer Inst.

[65] Detterbeck FC, Boffa DJ, Tanoue LT (2009). The new lung cancer staging system. Chest.

[66] Lee YJ, Choi HJ, Kim SK, Chang J, Moon JW, Park IK, Kim JH, Cho BC (2010). Frequent central nervous system failure after clinical benefit with epidermal growth factor receptor tyrosine kinase inhibitors in Korean patients with nonsmall-cell lung cancer. Cancer.

[67] Gow CH, Chien CR, Chang YL, Chiu YH, Kuo SH, Shih JY, Chang YC, Yu CJ, Yang CH, Yang PC (2008). Radiotherapy in lung adenocarcinoma with brain metastases: effects of activating epidermal growth factor receptor mutations on clinical response. Clin Cancer Res.

[68] Shukuya T, Takahashi T, Naito T, Kaira R, Ono A, Nakamura Y, Tsuya A, Kenmotsu H, Murakami H, Harada H, Mitsuya K, Endo M, Nakasu Y (2011). Continuous EGFR-TKI administration following radiotherapy for non-small cell lung cancer patients with isolated CNS failure. Lung Cancer.

[69] Hirano Y, Oda M, Tsunezuka Y, Ishikawa N, Watanabe G (2005). Long-term survival cases of lung cancer presented as solitary bone metastasis. Ann Thorac Cardiovasc Surg.

[70] Mercier O, Fadel E, de Perrot M, Mussot S, Stella F, Chapelier A, Dartevelle P (2005). Surgical treatment of solitary adrenal metastasis from non-small cell lung cancer. J Thorac Cardiovasc Surg.

[71] Park K, Yu CJ, Kim SW, Lin MC, Sriuranpong V, Tsai CM, Lee JS, Kang JH, Chan KC, Perez-Moreno P, Button P, Ahn MJ, Mok T (2016). First-Line Erlotinib Therapy Until Beyond Response Evaluation Criteria in Solid Tumors Progression in Asian Patients With Epidermal Growth Factor Receptor Mutation-Positive Non-Small-Cell Lung Cancer: The ASPIRATION Study. JAMA Oncol.

[72] Yu HA, Sima CS, Huang J, Solomon SB, Rimner A, Paik P, Pietanza MC, Azzoli CG, Rizvi NA, Krug LM, Miller VA, Kris MG, Riely GJ (2013). Local therapy with continued EGFR tyrosine kinase inhibitor therapy as a treatment strategy in EGFR-mutant advanced lung cancers that have developed acquired resistance to EGFR tyrosine kinase inhibitors. J Thorac Oncol.

[73] Gomez DR, Blumenschein GR, Lee JJ, Hernandez M, Ye R, Camidge DR, Doebele RC, Skoulidis F, Gaspar LE, Gibbons DL, Karam JA, Kavanagh BD, Tang C (2016). Local consolidative therapy versus maintenance therapy or observation for patients with oligometastatic non-small-cell lung cancer without progression after first-line systemic therapy: a multicentre, randomised, controlled, phase 2 study. Lancet Oncol.

[74] Oser MG, Niederst MJ, Sequist LV, Engelman JA (2015). Transformation from non-small-cell lung cancer to small-cell lung cancer: molecular drivers and cells of origin. Lancet Oncol.

[75] Yu HA, Arcila ME, Rekhtman N, Sima CS, Zakowski MF, Pao W, Kris MG, Miller VA, Ladanyi M, Riely GJ (2013). Analysis of tumor specimens at the time of acquired resistance to EGFR-TKI therapy in 155 patients with EGFR-mutant lung cancers. Clin Cancer Res.

[76] Kuo CH, Lin SM, Lee KY, Chung FT, Hsieh MH, Fang YF, Yu CT, Kuo HP (2010). Subsequent chemotherapy improves survival outcome in advanced non-small-cell lung cancer with acquired tyrosine kinase inhibitor resistance. Clin Lung Cancer.

[77] Tseng YH, Hung HY, Sung YC, Tseng YC, Lee YC, Whang-Peng J, Chen YM (2016). Efficacy of chemotherapy in epidermal growth factor receptor (EGFR) mutated metastatic pulmonary adenocarcinoma patients who had acquired resistance to first-line EGFR tyrosine kinase inhibitor (TKI). J Chemother.

[78] Soria JC, Wu YL, Nakagawa K, Kim SW, Yang JJ, Ahn MJ, Wang J, Yang JCH, Lu Y, Atagi S, Ponce S, Lee DH, Liu Y (2015). Gefitinib plus chemotherapy versus placebo plus chemotherapy in EGFR-mutation-positive non-small-cell lung cancer after progression on first-line gefitinib (IMPRESS): a phase 3 randomised trial. Lancet Oncol.

[79] Gandara DR, Li T, Lara PN, Kelly K, Riess JW, Redman MW, Mack PC (2014). Acquired resistance to targeted therapies against oncogene-driven non-small-cell lung cancer: approach to subtyping progressive disease and clinical implications. Clin Lung Cancer.

[80] Shukuya T, Takahashi T, Naito T, Kaira R, Ono A, Nakamura Y, Tsuya A, Kenmotsu H, Murakami H, Harada H, Mitsuya K, Endo M, Nakasu Y (2011). Continuous EGFR-TKI administration following radiotherapy for non-small cell lung cancer patients with isolated CNS failure. Lung Cancer.

[81] Weickhardt AJ, Scheier B, Burke JM, Gan G, Lu X, Bunn PA, Aisner DL, Gaspar LE, Kavanagh BD, Doebele RC, Camidge DR (2012). Local ablative therapy of oligoprogressive disease prolongs disease control by tyrosine kinase inhibitors in oncogene-addicted non-small-cell lung cancer. J Thorac Oncol.

[82] Gandara DR, Grimminger P, Mack PC, Lara PN, Li T, Danenberg PV, Danenberg KD (2010). Association of epidermal growth factor receptor activating mutations with low ERCC1 gene expression in non-small cell lung cancer. J Thorac Oncol.

[83] Lee JO, Kim TM, Lee SH, Kim DW, Kim S, Jeon YK, Chung DH, Kim WH, Kim YT, Yang SC, Kim YW, Heo DS, Bang YJ (2011). Anaplastic lymphoma kinase translocation: a predictive biomarker of pemetrexed in patients with non-small cell lung cancer. J Thorac Oncol.

[84] Miller VA, Hirsh V, Cadranel J, Chen YM, Park K, Kim SW, Zhou C, Su WC, Wang M, Sun Y, Heo DS, Crino L, Tan EH (2012). Afatinib versus placebo for patients with advanced, metastatic non-small-cell lung cancer after failure of erlotinib, gefitinib, or both, and one or two lines of chemotherapy (LUX-Lung 1): a phase 2b/3 randomised trial. Lancet Oncol.

[85] Reckamp KL, Giaccone G, Camidge DR, Gadgeel SM, Khuri FR, Engelman JA, Koczywas M, Rajan A, Campbell AK, Gernhardt D, Ruiz-Garcia A, Letrent S, Liang J (2014). A phase 2 trial of dacomitinib (PF-00299804), an oral, irreversible pan-HER (human epidermal growth factor receptor) inhibitor, in patients with advanced non-small cell lung cancer after failure of prior chemotherapy and erlotinib. Cancer.

[86] Janjigian YY, Smit EF, Groen HJM, Horn L, Gettinger S, Camidge DR, Riely GJ, Wang B, Fu Y, Chand VK, Miller VA, Pao W (2014). Dual inhibition of EGFR with afatinib and cetuximab in kinase inhibitor-resistant EGFR-mutant lung cancer with and without T790M mutations. Cancer Discov.

[87] Janjigian YY, Azzoli CG, Krug LM, Pereira LK, Rizvi NA, Pietanza MC, Kris MG, Ginsberg MS, Pao W, Miller VA, Riely GJ (2011). Phase I/II trial of cetuximab and erlotinib in patients with lung adenocarcinoma and acquired resistance to erlotinib. Clin Cancer Res.

[88] Ramalingam S, Forster J, Naret C, Evans T, Sulecki M, Lu H, Teegarden P, Weber MR, Belani CP (2008). Dual inhibition of the epidermal growth factor receptor with cetuximab, an IgG1 monoclonal antibody, and gefitinib, a tyrosine kinase inhibitor, in patients with refractory non-small cell lung cancer (NSCLC): a phase I study. J Thorac Oncol.

[89] Cross DA, Ashton SE, Ghiorghiu S, Eberlein C, Nebhan CA, Spitzler PJ, Orme JP, Finlay MR, Ward RA, Mellor MJ, Hughes G, Rahi A, Jacobs VN (2014). AZD9291, an irreversible EGFR TKI, overcomes T790M-mediated resistance to EGFR inhibitors in lung cancer. Cancer Discov.

[90] Walter AO, Sjin RT, Haringsma HJ, Ohashi K, Sun J, Lee K, Dubrovskiy A, Labenski M, Zhu Z, Wang Z, Sheets M, St Martin T, Karp R (2013). Discovery of a mutant-selective covalent inhibitor of EGFR that overcomes T790M-mediated resistance in NSCLC. Cancer Discov.

[91] Park K, Han JY, Kim DW, Bazhenova LA, Ou SH, Pang YK, Hin HS, Juan O, Son J, Jänne P (2016). 190TiP: ELUXA 1: Phase II study of BI 1482694 (HM61713) in patients (pts) with T790M-positive non-small cell lung cancer (NSCLC) after treatment with an epidermal growth factor receptor tyrosine kinase inhibitor (EGFR TKI). J Thorac Oncol.

[92] Lelais G, Epple R, Marsilje TH, Long YO, McNeill M, Chen B, Lu W, Anumolu J, Badiger S, Bursulaya B, DiDonato M, Fong R, Juarez J (2016). Discovery of (R, E)- N -(7-Chloro-1-(1-[4-(dimethylamino)but-2-enoyl]azepan-3-yl)-1 H -benzo[ d ]imidazol-2-yl)-2-methylisonicotinamide (EGF816), a Novel, Potent, and WT Sparing Covalent Inhibitor of Oncogenic (L858R, ex19del) and Resistant (T790M) EGFR Mutants for the Treatment of EGFR Mutant Non-Small-Cell Lung Cancers. J Med Chem.

[93] TAGRISSOTM (AZD9291) approved by the US FDA for patients with EGFR T790M mutation-positive metastatic non-small cell lung cancer. AstraZeneca. https://www.astrazeneca.com/media-centre/press-releases/2015/TAGRISSO-AZD9291-approved-by-the-US-FDA-for-patients-with-EGFR-T790M-mutation-positive-metastatic-non-small-cell-lung-cancer-13112015.html.

[94] Clifford R AstraZeneca wins European approval for Tagrisso: the first-in-class lung cancer treatment receives a conditional license. http://www.pmlive.com/pharma_news/astrazeneca_wins_european_approval_for_tagrisso_924068#.

[95] Stockaboo. Tagrisso approved in China as first-in-class treatment for EGFR T790M mutation-positive metastatic non-small cell lung cancer.

[96] Mok TS, Wu YL, Ahn MJ, Garassino MC, Kim HR, Ramalingam SS, Shepherd FA, He Y, Akamatsu H, Theelen WS, Lee CK, Sebastian M, Templeton A (2017). Osimertinib or Platinum–Pemetrexed in EGFR T790M–Positive Lung Cancer. N Engl J Med.

[97] Jänne PA, Yang JC, Kim DW, Planchard D, Ohe Y, Ramalingam SS, Ahn MJ, Kim SW, Su WC, Horn L, Haggstrom D, Felip E, Kim JH (2015). AZD9291 in EGFR Inhibitor–Resistant Non–Small-Cell Lung Cancer. N Engl J Med.

[98] Oxnard GR, Thress KS, Alden RS, Lawrance R, Paweletz CP, Cantarini M, Yang JC, Barrett JC, Jänne PA (2016). Association Between Plasma Genotyping and Outcomes of Treatment With Osimertinib (AZD9291) in Advanced Non-Small-Cell Lung Cancer. J Clin Onco.

[99] Yang J, Ramalingam SS, Jänne PA, Cantarini M, Mitsudomi T (2016). LBA2_PR: Osimertinib (AZD9291) in pre-treated pts with T790M-positive advanced NSCLC: updated Phase 1 (P1) and pooled Phase 2 (P2) results. J Thorac Oncol.

[100] Ramalingam S, Yang JC, Lee CK, Kurata T, Kim DW, John T, Nogami N, Ohe Y, Jänne PA (2016). LBA1_PR: Osimertinib as first-line treatment for EGFR mutation-positive advanced NSCLC: updated efficacy and safety results from two Phase I expansion cohorts. J Thorac Oncol.

[101] ClinicalTrial gov. AZD9291 Versus Gefitinib or Erlotinib in Patients With Locally Advanced or Metastatic Non-small Cell Lung Cancer (FLAURA). https://clinicaltrials.gov/ct2/show/NCT02296125.

[102] Ahn M, Yang J, Yu H, Saka H, Ramalingam S, Goto K, Kim S, Yang L, Walding A, Oxnard GR 136O - Osimertinib combined with durvalumab in EGFR-mutant non-small cell lung cancer: Results from the TATTON phase Ib trial. ESMO.

[103] Taylor P AZ halts durvalumab combination trials on safety grounds: Says move is a precautionary step after respiratory disease reports. http://www.pmlive.com/pharma_news/az_halts_durvalumab_combination_trials_on_safety_grounds_840199.

[104] Sequist LV, Soria JC, Camidge DR (2016). Update to Rociletinib Data with the RECIST Confirmed Response Rate. N Engl J Med.

[105] Broderick J Clovis Ends Development of Rociletinib in Lung Cancer. http://www.onclive.com/web-exclusives/clovis-ends-development-of-rociletinib-in-lung-cancer.

[106] Terry M Deaths Lead Boehringer Ingelheim to Cancel $730 Million Cancer Deal With Hanmi Pharma (128940.KS), Hanmi Stock Tanks. http://www.biospace.com/News/deaths-lead-boehringer-ingelheim-to-cancel-730/434245.

[107] Park K, Lee JS, Han JY, Lee KH, Kim JH, Cho EK, Cho JY, Min YJ, Kim JS, Kim DW (2016). 1300: Efficacy and safety of BI 1482694 (HM61713), an EGFR mutant-specific inhibitor, in T790M-positive NSCLC at the recommended phase II dose. J Thorac Oncol Off Publ Int Assoc Study Lung Cancer.

[108] Wu YL, Kim DW, Felip E, Zhang L, Liu X, Zhou CC, Lee DH, Han JY, Krohn A, Lebouteiller R, Xu C, Squires M, Akimov M, Tan DS (2016). Phase (Ph) II safety and efficacy results of a single-arm ph ib/II study of capmatinib (INC280) + gefitinib in patients (pts) with EGFR-mutated (mut), cMET-positive (cMET+) non-small cell lung cancer (NSCLC). J Clin Oncol.

[109] Neal JW, Dahlberg SE, Wakelee HA, Aisner SC, Bowden M, Huang Y, Carbone DP, Gerstner GJ, Lerner RE, Rubin JL, Owonikoko TK, Stella PJ, Steen PD (2016). Cabozantinib (C), erlotinib (E) or the combination (E+C) as second- or third-line therapy in patients with EGFR wild-type (wt) non-small cell lung cancer (NSCLC): A randomized phase 2 trial of the ECOG-ACRIN Cancer Research Group (E1512). Lancet Oncol.

[110] Tan DS, Lim KH, Tai SM, Ahmad A, Pan S, Ng QS, Ang MK, Gogna A, Ng YL, Tan BS, Lee HY, Krisna SS, Lau DP (2013). A phase Ib safety and tolerability study of a pan class I PI3K inhibitor buparlisib (BKM120) and gefitinib (gef) in EGFR TKI-resistant NSCLC. J Clin Oncol.

[111] Hwang KE, Jung JW, Oh SJ, Park MJ, Shon YJ, Choi KH, Jeong ET, Kim HR (2015). Transformation to small cell lung cancer as an acquired resistance mechanism in EGFR-mutant lung adenocarcinoma: a case report of complete response to etoposide and cisplatin. Tumori.

[112] Janne PA, Ahn MJ, Kim DW, Kim SW, Planchard D, Ramalingam SS, Frewer P, Cantarini M, Ghiorghiu S, Yang JC (2015). LBA3 * A phase i study of azd9291 in patients with egfr-tki-resistant advanced nsclc - updated progression free survival and duration of response data. Ann Oncol.

[113] Soria J, Kim S, Wu Y, Nakagawa K, Yang J, Ahn M, Wang J, Yang JC, Lu Y, Atagi S (2016). Ponce Aix S, Rukazenkov Y, Taylor R, Mok TS. Gefitinib/Chemotherapy vs Chemotherapy in EGFR Mutation-Positive NSCLC Resistant to First-Line Gefitinib: IMPRESS T790M Subgroup Analysis. Ann Oncol.

[114] Ettinger DS, Wood DE, Akerley W, Bazhenova LA, Borghaei H, Camidge DR, Cheney RT, Chirieac LR, D’Amico TA, Demmy TL, Dilling TJ, Dobelbower MC, Govindan R (2015). Non-Small Cell Lung Cancer, Version 6.2015. JNCCN.

[115] Lindeman NI, Cagle PT, Beasley MB, Chitale DA, Dacic S, Giaccone G, Jenkins RB, Kwiatkowski DJ, Saldivar JS, Squire J, Thunnissen E, Ladanyi M (2013). College of American Pathologists International Association for the Study of Lung Cancer, Association for Molecular Pathology. Molecular testing guideline for selection of lung cancer patients for EGFR and ALK tyrosine kinase inhibitors: guideline from the College of American Pathologists, International Association for the Study of Lung Cancer, and Association for Molecular Pathology. JMD.

[116] Masters GA, Temin S, Azzoli CG, Giaccone G, Baker S, Brahmer JR, Ellis PM, Gajra A, Rackear N, Schiller JH, Smith TJ, Strawn JR, Trent D (2015). Systemic Therapy for Stage IV Non-Small-Cell Lung Cancer: American Society of Clinical Oncology Clinical Practice Guideline Update. J Clin Oncol.

[117] Ettinger DS, Wood DE, Akerley W, Bazhenova LA, Borghaei H, Camidge DR, Cheney RT, Chirieac LR, D’Amico TA, Dilling TJ, Dobelbower MC, Govindan R, Hennon M (2016). NCCN Guidelines Insights: Non-Small Cell Lung Cancer, Version 4.2016. JNCCN.

[118] Punnoose EA, Atwal S, Liu W, Raja R, Fine BM, Hughes BG, Hicks RJ, Hampton GM, Amler LC, Pirzkall A, Lackner MR (2012). Evaluation of circulating tumor cells and circulating tumor DNA in non-small cell lung cancer: association with clinical endpoints in a phase II clinical trial of pertuzumab and erlotinib. Clin Cancer Res.

[119] Douillard JY, Ostoros G, Cobo M, Ciuleanu T, Cole R, McWalter G, Walker J, Dearden S, Webster A, Milenkova T, McCormack R (2014). Gefitinib treatment in EGFR mutated caucasian NSCLC: circulating-free tumor DNA as a surrogate for determination of EGFR status. J Thorac Oncol.

[120] Luo J, Shen L, Zheng D (2014). Diagnostic value of circulating free DNA for the detection of EGFR mutation status in NSCLC: a systematic review and meta-analysis. Sci Rep.

[121] Douillard JY, Ostoros G, Cobo M, Ciuleanu T, McCormack R, Webster A, Milenkova T (2014). First-line gefitinib in Caucasian EGFR mutation-positive NSCLC patients: a phase-IV, open-label, single-arm study. Br J Cancer.

[122] US Food and Drug Administration FDA approves first blood test to detect gene mutation associated with non-small cell lung cancer. https://www.fda.gov/NewsEvents/Newsroom/PressAnnouncements/ucm504488.htm.

[123] US Food and Drug Administration cobas EGFR Mutation Test v2. https://www.fda.gov/drugs/informationondrugs/approveddrugs/ucm504540.htm.

[124] Lee JY, Qing X, Xiumin W, Yali B, Chi S, Bak SH, Lee HY, Sun JM, Lee SH, Ahn JS, Cho EK, Kim DW, Kim HR (2016). Longitudinal monitoring of EGFR mutations in plasma predicts outcomes of NSCLC patients treated with EGFR TKIs: Korean Lung Cancer Consortium (KLCC-12-02). Oncotarget.

[125] Zheng D, Ye X, Zhang MZ, Sun Y, Wang JY, Ni J, Zhang HP, Zhang L, Luo J, Zhang J, Tang L, Su B, Chen G (2016). Plasma EGFR T790M ctDNA status is associated with clinical outcome in advanced NSCLC patients with acquired EGFR-TKI resistance. Sci Rep.

[126] Marchetti A, Palma JF, Felicioni L, De Pas TM, Chiari R, Del Grammastro M, Filice G, Ludovini V, Brandes AA, Chella A, Malorgio F, Guglielmi F, De Tursi M (2015). Early Prediction of Response to Tyrosine Kinase Inhibitors by Quantification of EGFR Mutations in Plasma of NSCLC Patients. J Thorac Oncol.

